# Acoustic Localisation as a Tool to Aid Monitoring of Golden Jackals (*Canis aureus*)

**DOI:** 10.1002/ece3.71041

**Published:** 2025-03-04

**Authors:** Bethany R. Smith, Elisabeth Bru, Mihaela Faur, Arik Kershenbaum

**Affiliations:** ^1^ School of Animal, Rural and Environmental Sciences Nottingham Trent University Southwell UK; ^2^ Institute of Zoology Zoological Society of London London UK; ^3^ Department of Life Sciences Imperial College London, Silwood Park Campus Ascot UK; ^4^ Fauna & Flora The David Attenborough Building Cambridge UK; ^5^ Girton College, and Department of Zoology University of Cambridge Cambridge UK

**Keywords:** animal communication, autonomous recording unit, conservation technology, multilateration, passive acoustic monitoring

## Abstract

Golden jackals ( *Canis aureus* ) have rapidly expanded their range across Europe, raising ecological and socioeconomic concerns. As a highly vocal species, jackals can be monitored using howl surveys or passive acoustic monitoring (PAM) to estimate population sizes and habitat preferences. A recent advancement in PAM is acoustic localisation, which estimates the source of sounds by measuring the time differences of their arrival at multiple synchronised recorders. This technique can improve the accuracy of population and density estimates by more precisely calculating distances between vocalising animals and recorders. However, GPS‐synchronised acoustic recorders are costly, which limits their broader use. In this study, we tested the efficacy of a low‐cost recorder, the CARACAL, for acoustic localisation of golden jackals. We deployed 10 CARACALs over a 10 km^2^ area of the Lunca Mureşului Natural Park, Romania, for seven nights. Alongside passively recording jackal howls, we also conducted howl surveys. We recorded jackal howls every night of the survey and successfully localised 27 jackal and 16 human howls, with human howls localised to within an average of 41 m of their actual location. The average distance between the recorders and estimated positions of vocalising jackals was just under 1 km, with howls detected from as far as 2.5 km away. However, some jackal howls were not detected clearly, or at all, on active recorders that were as little as 0.9 km away. Based on these results, we recommend a conservative spacing of 0.8–1 km between recorders in future deployments, though this will depend on local environmental conditions. Overall, this study highlights acoustic localisation as a valuable tool for improving monitoring efforts and gathering more detailed data on jackal ecology. This information could significantly contribute to understanding their expanding range across Europe while informing the development of effective monitoring and management strategies for golden jackals.

## Graphical abstract

In this study, we tested the low‐cost CARACAL recorder for acoustic localisation of golden jackals in Romania. We successfully detected and localised jackal howls, recommending an optimal recorder spacing of 0.8–1 km. These results highlight acoustic localisation as a valuable tool for enhancing jackal monitoring that could offer insights into their expanding European range and inform effective management strategies.

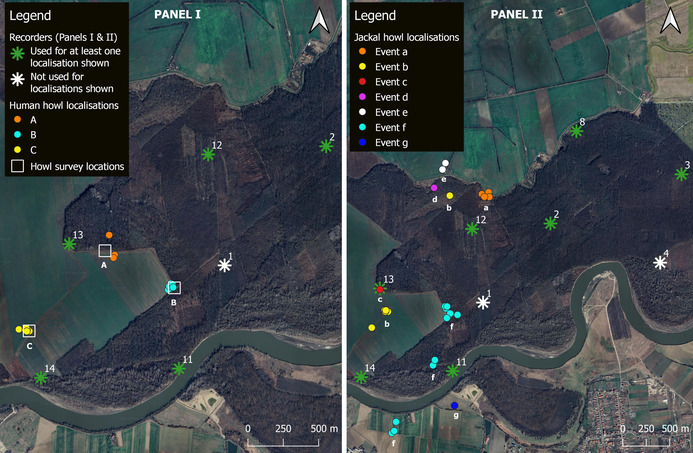

## 1 Introduction

The golden jackal ( *Canis aureus* ) is a widespread mesopredator native to Asia and Europe that has been exhibiting one of the fastest range expansions among mammals in Europe over the last few decades (Arnold et al. 2012; Trouwborst et al. 2015). Starting in south eastern Europe, jackals have been expanding northwards and westwards (Spassov and Acosta‐Pankov 2019; Cunze and Klimpel 2022), with a remarkable ability for long‐distance dispersal over thousands of kilometres (Rutkowski et al. 2015; Bogdanowicz et al. 2024). Jackals are now found across Europe, with records from as far west as Spain (Sáenz de Buruaga et al. 2023), as far north as the Russian Subarctic (Rykov et al. 2022) and even beyond the Arctic Circle in Norway and Finland (Sørensen and Larsø 2021; Kojola et al. 2024). The reasons for this dramatic range expansion are debated but are likely a combination of climate change (Fabbri et al. 2014; Cunze and Klimpel 2022), dietary flexibility (Lanszki et al. 2022) and adaptation to human‐dominated landscapes (Šálek et al. 2014; Fenton et al. 2021). Some studies theorise that mesopredator release might have also played a role, with historic persecution of wolves across Europe removing top‐down control of jackals (Krofel et al. 2017; Newsome et al. 2017; Wennink et al. 2019).

The rapid range expansion of jackals across Europe raises questions about both the ecological and social consequences. First, as an opportunistic mesopredator feeding on mostly small mammals (Lange et al. 2021), there is a risk of increased competitive interactions with other predators (Scheinin et al. 2006; Filacorda et al. 2021). Jackals are also facultative scavengers (Ćirović et al. 2016; Lange et al. 2021) and have been documented feeding off Eurasian lynx ( *Lynx lynx* ) kills, thus acting as a new kleptoparasite for lynx (Krofel et al. 2022). Some forecasts predict further increasing spatial overlap between these two species (Serva et al. 2023), which could increase the opportunity for interspecific competition, possibly to the detriment of threatened lynx populations (Krofel et al. 2022). However, there are accounts of jackals and red foxes ( *Vulpes vulpes* ) engaging in social interactions (Böcker et al. 2024), and some studies suggest that jackals can co‐occur with other European predators through niche partitioning (Tsunoda et al. 2018; Torretta et al. 2021; Guimarães et al. 2021).

The expansion of another predator in Europe is also potentially exacerbating existing human–wildlife conflicts as jackals are able to thrive in human‐dominated landscapes and are known to predate livestock and occasionally consume crops (Nemtzov and King 2001; Fanin et al. 2018; Srivathsa et al. 2019; Tănăsescu and Constantinescu 2019). In addition, jackals could be an important reservoir of parasites with high zoonotic potential for both animals and humans (Ionică et al. 2016; Gherman and Mihalca 2017; Mitková et al. 2017; Frey et al. 2022; Uiterwijk et al. 2023). However, studies have shown that some predation events attributed to jackals are caused by other species, such as red foxes (Schenekar et al. 2021), and that livestock found in jackal diets in southern Europe are mostly via scavenging of carcasses (Lange et al. 2021). In fact, jackals could provide key ecosystem services through their removal of rodent pests and scavenging of substantial amounts of animal waste, including wild boar ( *Sus scrofa* ) carcasses, which may help reduce the transmission of African swine fever (Ćirović et al. 2016; Probst et al. 2019; Kemenszky et al. 2022). Given the complex and largely unknown implications of jackal colonisation in new areas, it is imperative to monitor the spread of this species and to study the ensuing ecological and social impacts.

Several methods have been employed for monitoring jackals. These include using hunting bag data to infer population estimates (e.g., Szabó et al. 2009); surveying members of the public for sightings records (e.g., Ivanov et al. 2016); conducting sign surveys (including with the use of scat detection dogs) to detect jackal presence and estimate population size (e.g., Hatlauf, Böcker, et al. 2021); camera trapping to monitor jackal behaviours, distribution and estimate population densities (e.g., Krofel et al. 2022; Šprem et al. 2025); telemetry and GPS tracking of collared individuals to investigate spatial ecology (e.g., Fenton et al. 2021; Csányi et al. 2025); and genetic investigations into dispersal histories (Bogdanowicz et al. 2024; Stefanović et al. 2024). In addition, jackals are a highly vocal species that produce complex, long‐range howls used primarily in territorial interactions (Jaeger et al. 1996; Acosta‐Pankov et al. 2018). This behaviour facilitates their monitoring using acoustic methods, such as howl surveys, as they often vocally respond to either human howls or broadcasted jackal howls. Howl surveys have been used in many European countries to monitor jackals, alongside passive acoustic monitoring (PAM) (Šálek et al. 2014; Comazzi et al. 2016; Trbojević et al. 2018; Krofel et al. 2023). In PAM surveys, autonomous recording units (ARUs)—hereafter referred to as recorders—are deployed in the field and left for long periods of time to record spontaneous jackal howling. These acoustic methods are often used to infer group size, minimum population estimates and habitat preferences (Giannatos et al. 2005; Marques et al. 2013; Šálek et al. 2014; Selimovic et al. 2021).

The field of PAM is continuously advancing (Sugai et al. 2019; Gibb et al. 2019). One new development that is gaining traction in wildlife monitoring studies is acoustic localisation (Rhinehart et al. 2020). Acoustic localisation uses synchronised recorders to enable the calculation of differences in the time of arrival of the same sound at different recorders, which can then be used to estimate the location of the sound via multilateration (Mennill et al. 2012; Kershenbaum et al. 2019). Calculating more accurate distance measurements between jackals and recorders could help with estimations of density and population sizes (Marques et al. 2013; Graf and Hatlauf 2021). However, synchronised recorders are typically expensive, which limits their application and scalability. To overcome this, researchers recently developed a cheaper GPS‐synchronised recorder known as the CARACAL (Wijers et al. 2019). These recorders were first used in Zimbabwe to localise gunshots (to an average accuracy of 33.2 ± 15.3 m) and three wildlife species at distances greater than 1 km: Cape buffalo ( *Syncerus caffer* ), chacma baboon ( *Papio ursinus* ) and spotted hyena ( *Crocuta crocuta* ). Recently, we used these same recorders to detect and localise a range of canid species—grey wolves ( *Canis lupus* ), coyotes ( *Canis latrans* ) and domestic dogs ( *Canis familiaris* ) in the United States (Smith et al. 2021; Bru et al. 2023).

Given its previous efficacy at localising canid species, the aims of this study were to determine if the CARACAL could also be used for acoustic localisation of golden jackals and to provide the first steps in developing the optimal recorder deployment for acoustic localisation of this species. Systematic monitoring schemes of jackals are lacking across Europe (Papp et al. 2018; Hatlauf, Bayer, et al. 2021), so it is important to test and develop new, cost‐effective technologies. This will enable the improvement of monitoring efforts and gathering of information that can be used in early detection systems, wildlife management, and the studying of jackal ecology and behaviour.

## 2 Materials and Methods

### 2.1 Study Area and Deployment

**FIGURE 1:**
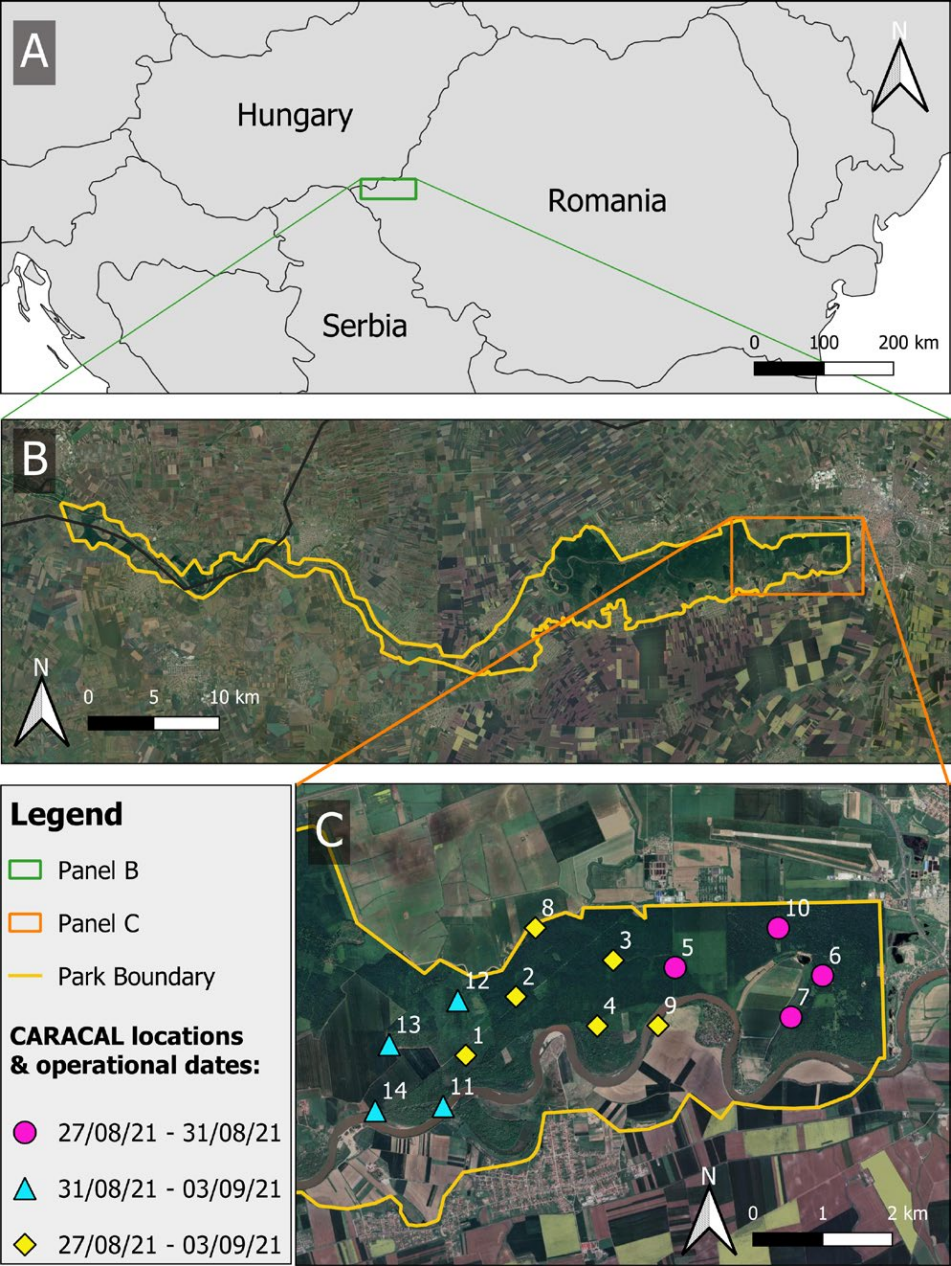
Locations and dates of recorder deployments in the Lunca Mureşului Natural Park, Romania. Maps produced in QGIS using Google Satellite imagery. The area to the left of Site 3 is the area shown in Figure 3.

The study was conducted in the Lunca Mureşului Natural Park, Romania (Figure 1). The Lunca Mureşului Natural Park is a 17,455 ha protected area in western Romania, stretching along the Mureș River along with the border with Hungary. The park is known for its diverse ecosystems, including riparian forests, wetlands and meadows, which provide habitats for a wide variety of wildlife, including rare reptiles and mammals, and over 200 bird species. Golden jackals have increased considerably across Romania since the 1980s (Banea et al. 2012; Farkas et al. 2017; Papp et al. 2018) and are known to be present in the park. Individuals in the park are likely to have originated from Hungary, where there has been an exponential increase in the jackal population since the mid‐1990s (Bijl et al. 2024). Apart from golden jackals, other medium and large terrestrial mammals found in the park include red deer ( *Cervus elaphus* ), wild boar, red fox, European otter ( *Lutra lutra* ) and Eurasian beaver ( *Castor fiber* ).

We deployed 10 CARACAL recorders across an approximately 10 km^2^ section of the eastern part of the park for seven nights in August–September 2021 (Figure 1). We selected deployment sites randomly in the field with a spacing of 800–1200 m between neighbouring recorders. After the first four nights, we moved four of the recorders further west for the final three nights, thus totalling 14 sites (Figure 1). This decision was taken as initial processing of the data in the field showed that a high proportion of jackal vocalisations were originating from further west in the park. All locations of the recorders are provided in Appendix 1: Table A1. The CARACALs have four fixed‐gain microphones and were factory programmed to record at a 44,100 Hz sample rate and 16‐bit resolution.

On three of the survey nights, we conducted howl surveys to elicit howling and ensure sufficient data were collected in the short duration of the study period. For these surveys, researchers howled rather than broadcasting recorded jackal howls. Upon arrival at each howl survey location, we turned off all lights and waited in silence for 5 min before one person stood apart from all others present and produced three singular howls in quick succession. If jackals did not respond, the researcher repeated this bout of three howls with 1–2 min between bouts, with up to three bouts at each location. After the third howling bout, we listened for up to 10 min in case of a delayed response (Comazzi et al. 2016). If no response was heard, we moved to the next location and repeated the process. If a response was heard, then we took note of the approximate compass direction of the howls and whether responses originated from one or more locations. We selected howling sites from across the deployment area and optimised sound transmission by howling at field margins and forest clearings. We howled from sites both inside and outside the convex hull of the recorders as this relates to how accurately sounds can be localised (Kershenbaum et al. 2019; Wijers et al. 2019).

### 2.2 Data Processing and Analysis

**FIGURE 2:**
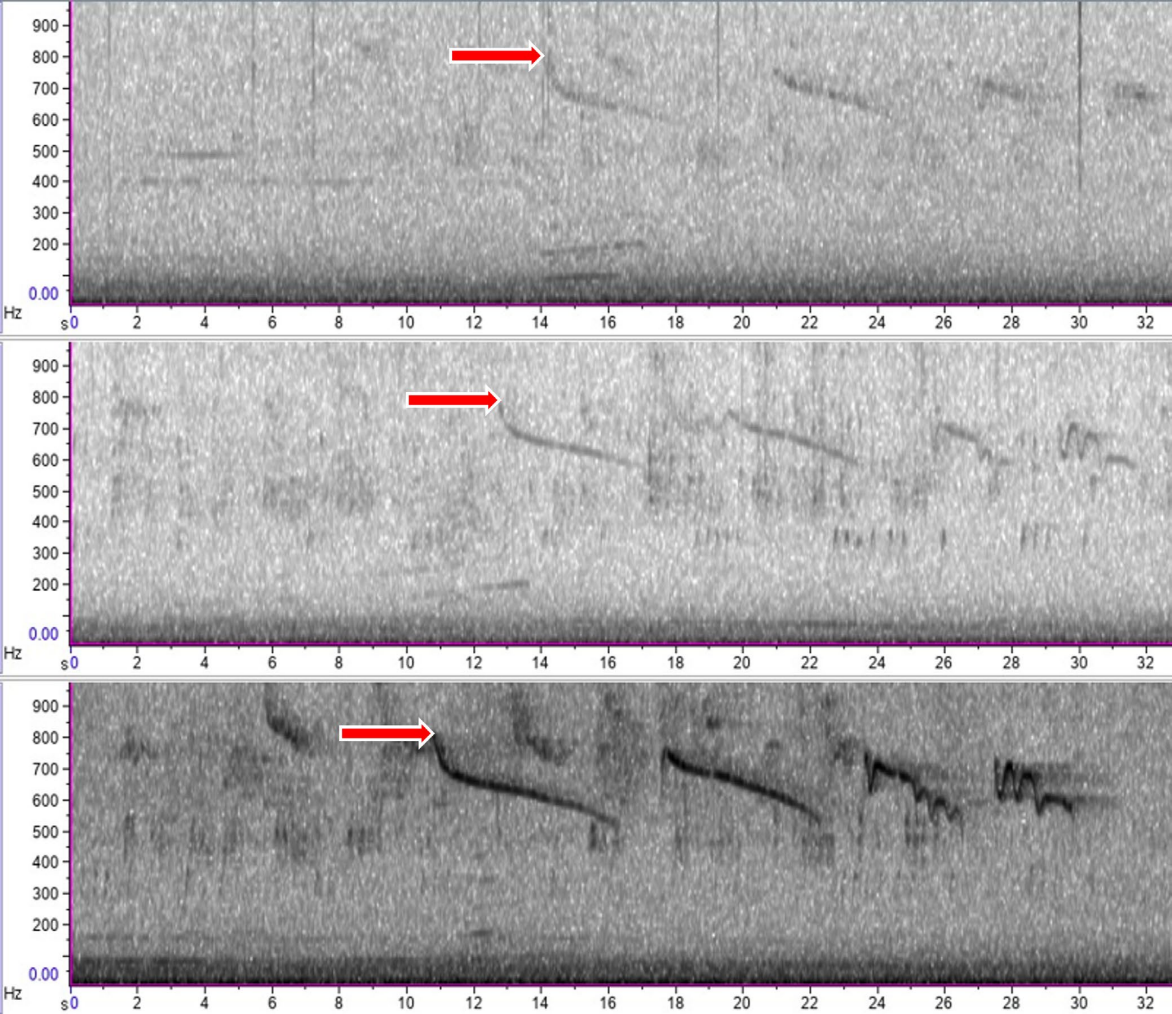
Example spectrograms showing a golden jackal vocalisation detected on three different CARACAL recorders. The salient points—the same points in the same howl across multiple recorders—are marked with red arrows. The sound is clearest (and loudest) on the bottom panel and arrives at this recorder first, indicating that the vocalising jackal was likely closest to this recorder relative to those in the middle and top spectrogram.

We downloaded all data from the CARACALs and synchronised the audio recordings, using the GPS timestamps present in the recording filenames. We visualised the data as spectrograms in Raven Pro 1.6 (Cornell Lab of Ornithology, Ithaca, NY) and identified any clear jackal and human vocalisations according to methods in Kershenbaum et al. (2019) and Smith et al. (2021). We then compiled multitrack spectrograms of howling events. We defined events as starting from the beginning of either human or jackal vocalisations until there was a period of silence of 1 min or more between subsequent calls. For example, Figure 2 shows one jackal howling event composed of four distinct howls because these were separated by less than 1 min. Within the multitrack files, we manually marked salient points, which are distinctive points of the same sound recorded on multiple devices (Figure 2). Previous experience has shown that automatic spectrogram cross‐correlation provides inferior results to manual matching due to the low signal‐to‐noise ratio of the howls (Kershenbaum et al. 2019). From within the same howling event, we marked salient points in multiple howls where possible but only marked one salient point per distinct howl. By zooming in on the time and frequency axes, we attempted to mark salient points with a time accuracy of about 50 ms (equivalent to about 15 m path length difference). We only marked salient points where we were confident from the howl characteristics that it was the same point in the same howl across the recorders. We also made a note as to whether the howls seemed to originate from the same or different locations based on the intensity of the howls and on which recorders they were detected. We then used the time differences of arrival between the salient points marked on three or more recorders to perform multilateration to locate both the human and jackal howls using published MATLAB scripts (MathWorks Inc.; Kershenbaum et al. 2019).

To first check the accuracy of the localisations, we calculated the Euclidean distance from the estimated positions of the human howls to the known locations that we howled from. We then measured the Euclidean distance from both the true human howling locations and the estimated jackal locations to the recorders on which these howls were detected and the recorders that were active but did not detect the same howls. We refer to howls as being detected on recorders if they were recorded sufficiently clearly to mark salient points, and not detected on recorders if either no howls were visible in the spectrograms or if salient points could not be confidently marked (i.e., the recording was of too poor quality) even though parts of the howls might actually have been recorded.

We also determined whether the howls were localised to sites inside or outside the local convex hull of the recorders that were used for the multilateration of each howl. Where jackal howls from the same event were localised to multiple locations, we cross‐referenced with our howl survey notes to see if we heard jackals respond from multiple directions. We also checked on the spectrograms whether these howls seemed to be from different directions by looking at the intensity of the sounds and the combinations of recorders on which they were detected.

## 3 Results

We recorded jackal howls on every night of the study period, predominantly between 6 pm and 6 am example: (see example of recorded jackal howls in Video 1).

**VIDEO 1:** Example spectrogram and audio of jackal howls that was recorded by a CARACAL during the study. Video content can be viewed at https://onlinelibrary.wiley.com/doi/10.1002/ece3.71041

**FIGURE 3:**
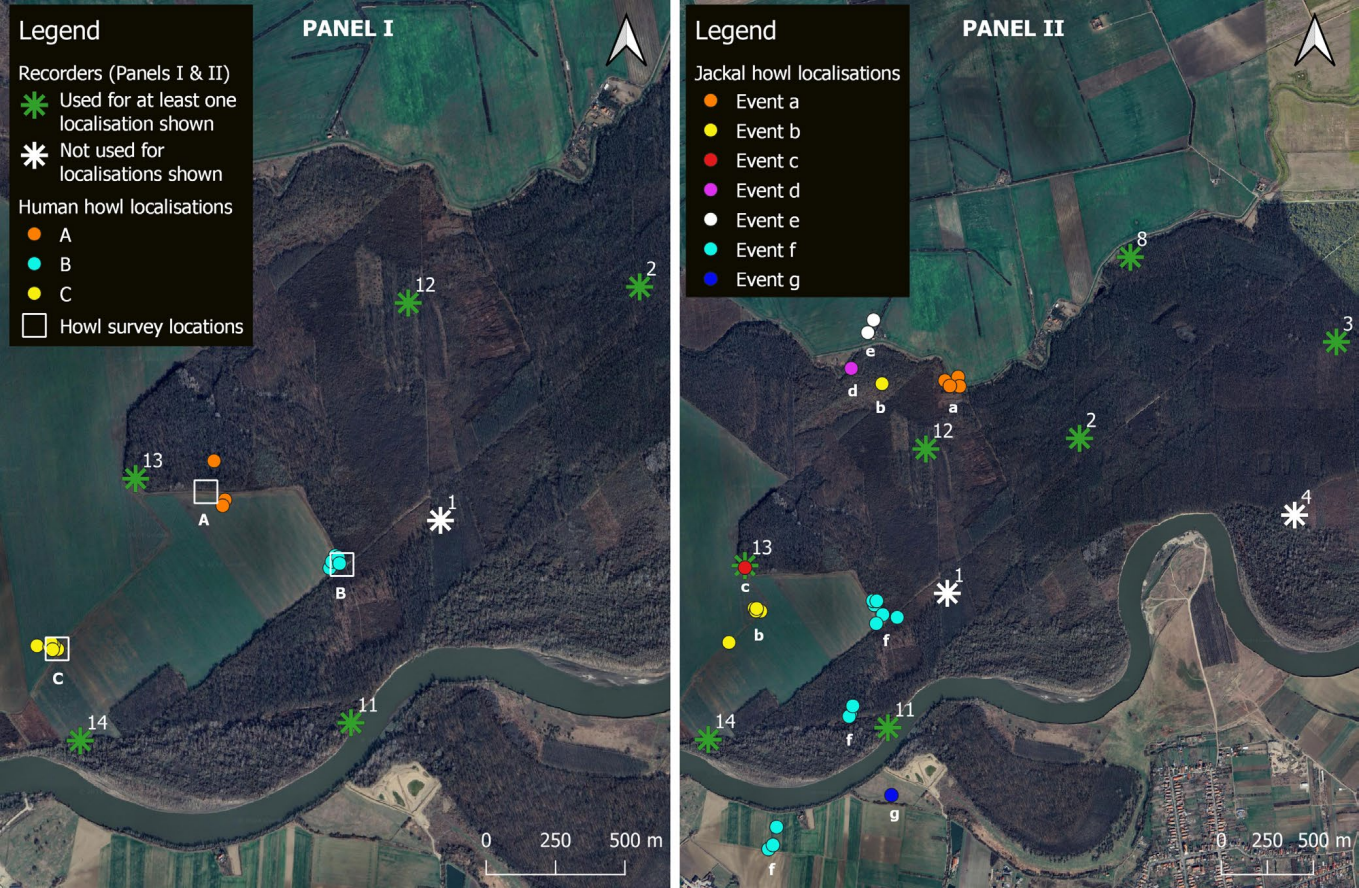
Panel I: Estimated positions of the 16 localised human howls (circles) relative to the three (A–C) howl survey sites (white squares). Panel II: Estimated positions of the 27 localised jackal howls (circles) grouped into seven events (a–f) whereby events are distinguished by a minute or more of silence between vocalisations. Events ‘b’ and ‘f’ show different jackals howling from two and three different locations, respectively. Recorder locations are denoted as green and white stars and labelled with the site number. Not all recorders shown were used for every localisation: those in green were used in at least one localisation and those in white were not used in any. The recorders shown in green in each panel are all of the recorders that contributed data to the localisations. Maps produced in QGIS using Google Satellite imagery.

Due to a technical malfunction that affected different recorders on different days, not all audio recordings could be synchronised so not all data from all devices could be used for acoustic localisation. In total, there were 35 multitrack files of single howling events where we could confidently identify jackal howls and 26 where we could confidently identify human howls. From these, seven events of human howling and seven events of jackal howling were recorded sufficiently clearly to mark salient points on three or more devices. Within these events, 16 human howls from three howling survey sites and 27 jackal howls were localised (Figure 3). Specifically, five human howls were localised using salient points marked across four recorders and 11 from salient points marked across three recorders. Seven jackal howls were localised using salient points marked across four recorders and 20 from salient points marked across three recorders. The subset of recorders that contributed to each localisation is provided in Appendix 1: Table A2. All of the localisations were estimated from salient points marked on recordings from seven locations (Sites 2, 3, 8, 11, 12, 13 and 14 in Figures 1 and 3).

**FIGURE 4:**
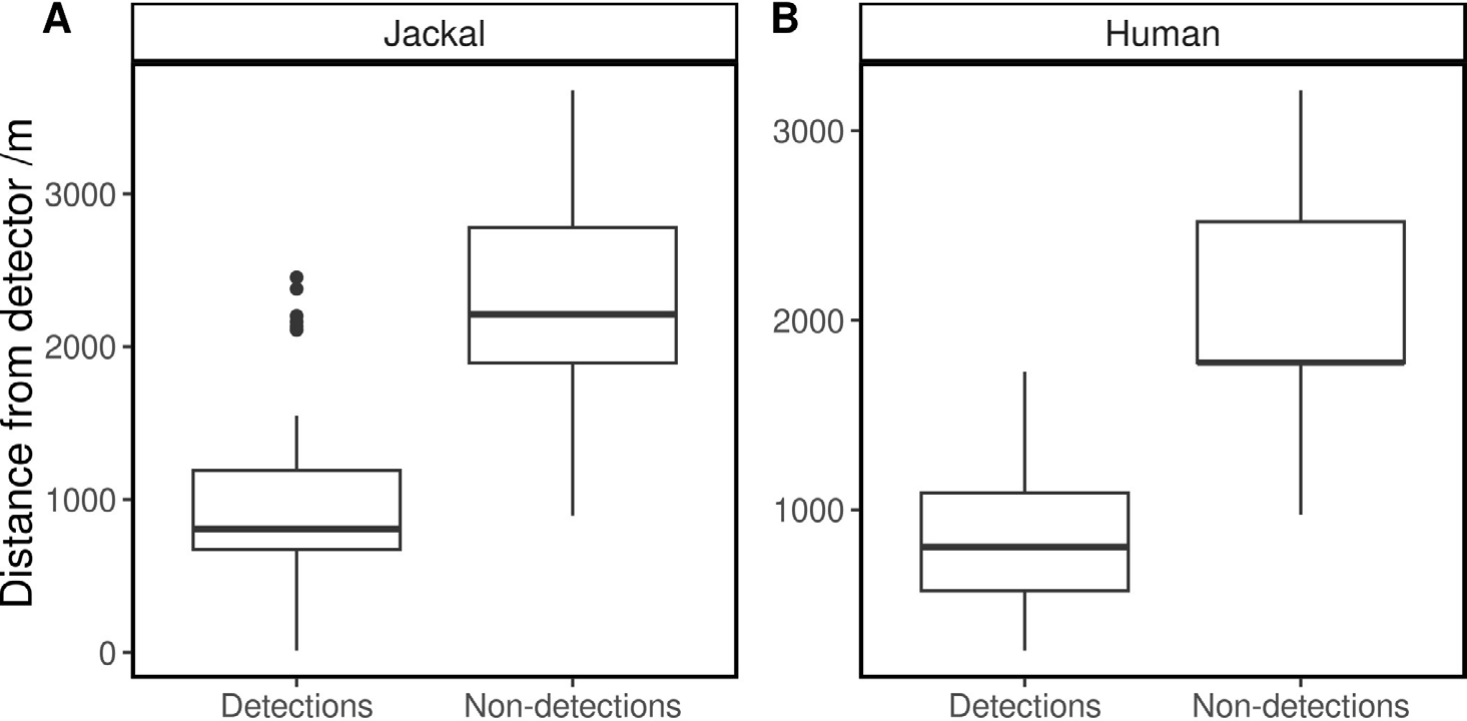
Distances of (A) estimated positions of localised jackal howls and (B) true locations of human howls from the recorders with which the howl was localised (detections). Also shown are the distances to the recorders that were active at the time of the howl but on which the howl was not detected (non‐detections).

Human howls were localised to an average of 41 m (range: 2–116 m) from the real location (Table 1) and were detected clearly enough to mark salient points on recorders up to 1.73 km away. Jackal howls were localised to points ranging from 11 m to 2.45 km (mean: 0.94 km) away from the recorders on which they were detected. For the 27 localised jackal howls, the mean maximum distance from the furthest recorder on which each howl was detected was 1.46 km (range: 0.91–2.45 km). However, there was some overlap in the distances of the recorders on which howls were detected and were not detected, with multiple non‐detections on recorders that were between 0.89 km and 3.68 km away from localised jackal howls (Figure 4), and 10 instances of howls not being detected on recorders that were closer to the howling individual than the furthest recorder on which the sound was detected (Appendix 1: Table A2). There was also overlap between the ranges of detection and non‐detection distances for human howls (Figure 4).

**TABLE 1:** Summary of localised human howls with the accuracy of the localisation, that is, the distance between the real location that the howl originated from (howl survey location) and the position that was estimated via acoustic localisation. The recorder sites that contributed to the localisation are given, along with whether the estimated position from the localisation fell inside or outside the local hull of the recorders that contributed to the localisation. The group refers to howl survey locations as depicted in Figure 3.

Howl no.	Date and time	Howl survey location	Estimated position by localisation	Distance (m)	Recorder sites	Local hull	Group
1	31/08/2021 20:29:05	46.14972, 21.18869	46.14945, 21.18958	75	2, 12, 13, 14	Inside	A
2	31/08/2021 20:30:11	46.14972, 21.18869	46.15072, 21.18907	115	12, 13, 14	Inside	A
3	31/08/2021 20:30:17	46.14972, 21.18869	46.14927, 21.18947	78	12, 13, 14	Inside	A
4	02/09/2021 22:38:35	46.14735, 21.19503	46.14763, 21.19471	40	11, 12, 13, 14	Inside	B
5	02/09/2021 22:38:47	46.14735, 21.19503	46.14723, 21.19445	46	11, 13, 14	Outside	B
6	02/09/2021 22:38:51	46.14735, 21.19503	46.14753, 21.19487	24	11, 12, 13, 14	Inside	B
7	02/09/2021 22:42:35	46.14735, 21.19503	46.14745, 21.19456	38	11, 12, 13, 14	Inside	B
8	02/09/2021 22:42:43	46.14735, 21.19503	46.14758, 21.19485	29	11, 12, 13, 14	Inside	B
9	02/09/2021 22:42:57	46.14735, 21.19503	46.14738, 21.19491	10	11, 13, 14	Outside	B
10	02/09/2021 22:57:41	46.14463, 21.18173	46.14462, 21.18175	2	11, 13, 14	Outside	C
11	02/09/2021 22:57:54	46.14463, 21.18173	46.14473, 21.18079	73	11, 13, 14	Outside	C
12	02/09/2021 22:57:59	46.14463, 21.18173	46.14471, 21.18139	28	11, 13, 14	Outside	C
13	02/09/2021 22:59:55	46.14463, 21.18173	46.14476, 21.18156	20	11, 13, 14	Outside	C
14	02/09/2021 23:00:02	46.14463, 21.18173	46.14471, 21.18139	28	11, 13, 14	Outside	C
15	02/09/2021 23:00:14	46.14463, 21.18173	46.14483, 21.18145	31	11, 13, 14	Outside	C
16	02/09/2021 23:02:06	46.14463, 21.18173	46.14461, 21.18152	16	11, 13, 14	Outside	C

Approximately half of the howls were localised to points outside the local convex hull of the recorders (human howls: *n* = 9; jackal howls: *n* = 14) and half inside (human howls: *n* = 7; jackal howls: *n* = 13). In two instances, jackal howls from the same event were localised to two and three different locations (Figure 4). We confirmed these were likely to be different groups of jackals howling because we noted jackal responses from multiple directions in the field, and they were detected on different combinations of recorders. All distance measurements are provided in Appendix 1: Table A2.

## 4 Discussion

This study is the first to demonstrate that acoustic localisation of golden jackals with GPS‐synchronised, low‐cost autonomous recorders is feasible. As such, this method offers a promising, cost‐effective addition to the suite of jackal monitoring techniques that could be implemented over large areas. Canid howling rates, including those of jackals, are known to vary seasonally with more frequent vocalisations around the mating season (Jaeger et al. 1996; Acosta‐Pankov et al. 2018). As such, acoustic monitoring should not be used instead of, but as a complement to, current survey methods such as hunting bag censuses, track and scat surveys, and camera trapping throughout the year.

We successfully localised human howls to an average of 41 m from their true locations, despite half of these locations being outside the local convex hulls of the recorders where accuracy is typically reduced (Kershenbaum et al. 2019; Wijers et al. 2019). This supports the reliability of our jackal howl localisations, despite their positions being unverifiable. We acknowledge that the accuracy of the human and jackal howl localisations is not strictly comparable due to some howls occurring in different areas and being detected on different subsets of recorders. However, we can be reasonably confident in the accuracy of jackal localisation and the associated distance measurements as many of the recorder subsets were the same (Appendix 1: Table A2), the habitat type across the study area is similar, and many of the estimated locations of howls in the same event cluster together (Figure 3), even when locations in the same event are being localised inside and outside the local convex hull of the recorders (Table 1; Appendix 1: Table A2). The average distance between the recorders on which the sound was detected and the howling individual was just under 1 km, with howls being detected as far as 2.5 km away. However, some localised howls were not detected on active recorders that were as little as 0.89 km away. Thus, we recommend a conservative spacing of recorders for acoustic localisation of jackals with CARACALs (or recorders of similar specifications) of 0.8–1 km. Although howls can be recorded from several kilometres away, this spacing would help ensure that howls are detected clearly on enough recorders for multilateration. This provides some improvement on previous studies using AudioMoth recorders (Hill et al. 2018) that found reliable distance estimations using relative sound level were possible up to only 400 m away (Graf and Hatlauf 2021).

However, we observed cases where recorders did not record howls clearly, or at all, despite being closer to the howling individual than recorders on which the howl was detected. This highlights how there are many factors influencing the probability of detection of vocalisations by recorders. The distance that vocalisations travel is likely to be affected by weather conditions and habitat variables, both of which vary by season. Weather conditions, including rain, humidity, wind speed and temperature, directly affect the acoustic properties of air, which is known to affect acoustic detection of animal vocalisations (Hannah 2006; Goerlitz 2018). Habitat variables such as understorey vegetation and tree structure also impact acoustic propagation and affect detection probability on acoustic recorders (Darras et al. 2016; Priyadarshani et al. 2018). As our surveys were conducted over a limited time in a single season, our results are not generalisable across the year. As such, our suggested spacing should be treated as a guideline that can be adjusted according to the season and habitat type, with more studies needed to address the optimal spacing of recorders under different environmental conditions.

As well as increasing the distance over which jackals can be detected by using more sensitive recorders, acoustic localisation brings many additional benefits. Imprecise distance estimates can lead to inaccurate density estimates (Yip et al. 2020); hence, knowing the location of vocalising jackals enables more accurate population estimates, which are needed to inform management actions. Additionally, acoustic localisation provides clearer insights into the number of jackal groups in an area. For example, we confirmed through acoustic localisation that jackals were howling from multiple locations at the same time. Without the localisation, howls detected from different groups of jackals in different but equidistant locations from a non‐GPS–synchronised recorder could be at similar intensities on a spectrogram and therefore look to be the same group howling. Thus, acoustic localisation can help to determine the number of individuals in a location and ultimately how well‐established jackal populations are in newly colonised areas across Europe. Furthermore, distinguishing between established groups and dispersing lone individuals could be useful for management purposes that aim to lethally remove individuals without disrupting social groups (Nemtzov and King 2001).

The identification of multiple groups howling at the same time from different places highlights a significant advantage of acoustic localisation for the study of vocal behaviour. This could permit studies of behavioural interactions between individuals and groups of jackals, as well as between jackals and other vocal species. One particularly vocal species recovering and now widespread across Europe is the grey wolf, an apex predator likely exerting top‐down pressure on golden jackals (Mohammadi et al. 2017; Krofel et al. 2017). On the contrary, there are reports of jackals and wolves hybridising (Kazimirov et al. 2024). There are also reports of jackals hybridising with domestic dogs (Galov et al. 2015; Ninausz et al. 2023; Stefanović et al. 2024), which is of particular interest in countries such as Romania, that have a large free‐ranging and feral dog population as well as widespread use of livestock guarding dogs. In fact, in this study, we observed jackals with atypical morphologies, potentially indicative of jackal–dog hybridisation in the study area—though not always (Barash et al. 2023)—and we recorded many instances of dogs seemingly barking in response to jackals howling. Some studies have even suggested that dogs, as well as wolves, might suppress golden jackals (Tsunoda et al. 2018). Thus, there is potential for a complex communication network between these sympatric canid species, as previously found for wolves, coyotes and dogs in the United States (Root‐Gutteridge et al. 2024), which could be explored with acoustic localisation studies.

Considering studying vocal communication between species, fine‐scale habitat preferences can also be investigated with acoustic localisation (Bru et al. 2023). The fine‐scale spatial ecology of golden jackals is little studied compared to their large‐scale distribution patterns (Fenton et al. 2021). Hence, acoustic localisation could also be used as a non‐invasive alternative, or complement, to telemetry and GPS‐tracking studies to better understand movement behaviour and resource selection. Although we currently have too few howl localisations from this study to draw any quantitative inferences regarding habitat preferences, jackals were localised to multiple habitat types within the park and surrounding area, including being close to anthropogenic features in the landscape. Whilst this is unsurprising given their nature as an opportunistic mesopredator that can adapt to human landscapes (Ćirović et al. 2016; Lange et al. 2021; Fenton et al. 2021), future studies could employ acoustic localisation to investigate fine‐scale jackal habitat preferences and dispersal dynamics in more detail, which in turn could be input into habitat suitability models (Wennink et al. 2019; Torretta et al. 2020). Such information could be useful to predict new areas of colonisation and potential hotspot areas for human–jackal conflicts.

Furthermore, habitat preferences inferred from acoustic localisation could be used to study interactions between jackals and other co‐occurring species. For example, it has been suggested by many that wolves might limit the distribution of jackals and that the two species are largely segregated in space, with wolves occurring in forested and mountainous areas farther from humans, while jackals more commonly found in agricultural areas and lowlands near human settlements (Trbojević et al. 2018; Wennink et al. 2019; Shakarashvili et al. 2020; Kraševec et al. 2021; Karamanlidis et al. 2023). Acoustic localisation data could help to better understand this relationship, especially where the sympatric occurrence of wolves and jackals is reportedly increasing (Shakarashvili et al. 2020; Guimarães et al. 2021).

As jackals continue to spread and increase in numbers across Europe, and with the ecological and socioeconomic consequences of this range expansion little understood, it is imperative that monitoring efforts are increased where possible and even integrated into national monitoring schemes as well as transboundary management plans for golden jackals (Papp et al. 2018; Hatlauf, Bayer, et al. 2021). Overall, despite its small scale, this study highlights acoustic localisation as a promising additional tool to increase the efficacy of monitoring as well as the amount of information gathered from acoustic surveys to overall better understand jackal ecology and movement across Europe.

## Author Contributions

**Bethany R. Smith:** conceptualization (equal), data curation (equal), formal analysis (equal), investigation (equal), methodology (equal), project administration (equal), visualization (lead), writing – original draft (lead). **Elisabeth Bru:** conceptualization (equal), data curation (equal), formal analysis (equal), investigation (equal), methodology (equal), project administration (equal), visualization (supporting), writing – original draft (supporting). **Mihaela Faur:** project administration (equal), writing – review and editing (equal). **Arik Kershenbaum:** conceptualization (equal), data curation (equal), funding acquisition (lead), methodology (equal), project administration (supporting), resources (lead), software (lead), supervision (lead), writing – review and editing (equal).

## Conflicts of Interest

The authors declare no conflicts of interest.

## Data Availability

All recorder locations are provided in Appendix 1: Table A1, and all localisations, including distances from the recorders to the estimated howl locations and howl survey locations, are provided in Table 1 and Appendix 1: Table A2.

## Appendix 1

**TABLE A1:** Locations of the 10 CARACALs and the deployment start and end times at each site.

Site number	CARACAL ID	Latitude	Longitude	Start	End
1	1	46.14877	21.19963	27/08/21 14:37:00	03/09/21 09:01:00
2	2	46.15633	21.20895	27/08/21 15:05:00	03/09/21 08:50:00
3	3	46.16101	21.22703	27/08/21 15:39:00	03/09/21 08:24:00
4	4	46.15255	21.22406	27/08/21 19:11:00	03/09/21 09:26:00
5	5	46.16007	21.23852	27/08/21 19:49:00	31/08/21 08:00:00
6	6	46.15904	21.26593	27/08/21 17:47:00	31/08/21 08:09:00
7	7	46.15368	21.26006	27/08/21 18:15:00	31/08/21 07:55:00
8	8	46.16518	21.21256	27/08/21 20:56:00	03/09/21 09:45:00
9	9	46.15261	21.23535	27/08/21 19:26:00	03/09/21 12:11:00
10	10	46.16516	21.25768	27/08/21 21:03:00	31/08/21 07:45:00
11	5	46.14221	21.19543	31/08/21 18:47:00	03/09/21 09:05:00
12	6	46.15583	21.19815	31/08/21 19:47:00	03/09/21 09:15:00
13	7	46.15015	21.18541	31/08/21 20:06:00	03/09/21 08:36:00
14	10	46.14165	21.1828	31/08/21 19:36:00	03/09/21 08:51:00

**TABLE A2:** Localised howls of both golden jackals (J) and humans (H). Lat‐L and Lon‐L are the position estimates of the localised howls, whereas Lat‐R and Lon‐R are the locations where the howl survey took place from. The groupings depicted in Figure 3 are given—for humans, these correspond to the different locations of the howling survey, whereas for jackals these correspond to the individual ‘events’ where vocalisations are separated by at least 1 min. The Local hull column states whether the estimated positions of the howls fell inside or outside the local convex hull of the recorders on which the howl was detected. Distances from the localised jackal howls and the real howl survey locations to each recorder site (S1–S14) are given. Recorders on which the sound was detected and therefore contributed to the localisation are highlighted in green, with the furthest recorder from the localised howl position denoted in a darker green. Recorders that were active but on which the sound was not detected are highlighted in pink. Where these pink values are also in bold and underlined, this denotes that the recorder was closer to the howling individual than the furthest recorder on which the sound was detected sufficiently clearly to mark salient points. Where the distance to the recorder is denoted as NA, this means that the device was either inactive at the time of the howl or there was a technical error with the GPS synchronisation, meaning that the data could not be used for localisation.

Howl no.	Species	Date and time	Figure 3 group	Local hull	Lat‐L	Lon‐L	Lat‐R	Lon‐R	S1	S2	S3	S4	S5	S6	S7	S8	S9	S10	S11	S12	S13	S14
1	H	31/08/2021 20:29:05	A	In	46.149453	21.189580	46.14972	21.18869	NA	1727.0	3212.5	NA	NA	NA	NA	2519.8	NA	NA	NA	997.4	257.4	1006.7
2	H	31/08/2021 20:30:11	A	In	46.150720	21.189071	46.14972	21.18869	NA	1727.0	3212.5	NA	NA	NA	NA	2519.8	NA	NA	NA	997.4	257.4	1006.7
3	H	31/08/2021 20:30:17	A	In	46.149266	21.189468	46.14972	21.18869	NA	1727.0	3212.5	NA	NA	NA	NA	2519.8	NA	NA	NA	997.4	257.4	1006.7
4	H	02/09/2021 22:38:35	B	In	46.147633	21.194712	46.14735	21.19503	NA	NA	NA	NA	NA	NA	NA	NA	NA	NA	573.0	974.2	804.7	1136.8
5	H	02/09/2021 22:38:47	B	Out	46.147233	21.194454	46.14735	21.19503	NA	NA	NA	NA	NA	NA	NA	NA	NA	NA	573.0	**974.2**	804.7	1136.8
6	H	02/09/2021 22:38:51	B	In	46.147531	21.194869	46.14735	21.19503	NA	NA	NA	NA	NA	NA	NA	NA	NA	NA	573.0	974.2	804.7	1136.8
7	H	02/09/2021 22:42:35	B	In	46.147445	21.194559	46.14735	21.19503	NA	NA	NA	NA	NA	NA	NA	NA	NA	NA	573.0	974.2	804.7	1136.8
8	H	02/09/2021 22:42:43	B	In	46.147581	21.194854	46.14735	21.19503	NA	NA	NA	NA	NA	NA	NA	NA	NA	NA	573.0	974.2	804.7	1136.8
9	H	02/09/2021 22:42:57	B	Out	46.147382	21.194915	46.14735	21.19503	NA	NA	NA	NA	NA	NA	NA	NA	NA	NA	573.0	**974.2**	804.7	1136.8
10	H	02/09/2021 22:57:41	C	Out	46.144621	21.181754	46.14463	21.18173	NA	NA	NA	NA	NA	NA	NA	NA	NA	NA	1090.5	1777.1	676.9	341.8
11	H	02/09/2021 22:57:54	C	Out	46.144735	21.180792	46.14463	21.18173	NA	NA	NA	NA	NA	NA	NA	NA	NA	NA	1090.5	1777.1	676.9	341.8
12	H	02/09/2021 22:57:59	C	Out	46.144706	21.181388	46.14463	21.18173	NA	NA	NA	NA	NA	NA	NA	NA	NA	NA	1090.5	1777.1	676.9	341.8
13	H	02/09/2021 22:59:55	C	Out	46.144759	21.181555	46.14463	21.18173	NA	NA	NA	NA	NA	NA	NA	NA	NA	NA	1090.5	1777.1	676.9	341.8
14	H	02/09/2021 23:00:02	C	Out	46.144706	21.181388	46.14463	21.18173	NA	NA	NA	NA	NA	NA	NA	NA	NA	NA	1090.5	1777.1	676.9	341.8
15	H	02/09/2021 23:00:14	C	Out	46.144829	21.181454	46.14463	21.18173	NA	NA	NA	NA	NA	NA	NA	NA	NA	NA	1090.5	1777.1	676.9	341.8
16	H	02/09/2021 23:02:06	C	Out	46.144611	21.181518	46.14463	21.18173	NA	NA	NA	NA	NA	NA	NA	NA	NA	NA	1090.5	1777.1	676.9	341.8
17	J	31/08/2021 21:18:19	a	Out	46.159345	21.200427	NA	NA	NA	737.9	2059.6	NA	NA	NA	NA	1138.9	NA	NA	NA	428.8	1545.5	2393.3
18	J	31/08/2021 21:18:24	a	Out	46.158892	21.200529	NA	NA	NA	709.2	2056.9	NA	NA	NA	NA	1162.1	NA	NA	NA	387.1	1518.7	2356.6
19	J	31/08/2021 21:18:40	a	Out	46.159167	21.199501	NA	NA	NA	794.1	2132.5	NA	NA	NA	NA	1209.1	NA	NA	NA	385.8	**1479.3**	2336.9
20	J	31/08/2021 21:18:48	a	Out	46.158908	21.199847	NA	NA	NA	758.3	2109.0	NA	NA	NA	NA	1203.4	NA	NA	NA	366.8	**1479.9**	2327.9
21	J	31/08/2021 21:26:46	b	In	46.148056	21.186057	NA	NA	NA	1991.2	3473.1	NA	NA	NA	NA	2794.7	NA	NA	NA	1272.2	238.3	756.1
22	J	31/08/2021 21:26:49	b	In	46.147919	21.186508	NA	NA	NA	1967.7	3447.9	NA	NA	NA	NA	2779.9	NA	NA	NA	1257.6	262.4	754.2
23	J	31/08/2021 21:26:52	b	In	46.147930	21.186177	NA	NA	NA	1989.6	3470.5	NA	NA	NA	NA	2797.6	NA	NA	NA	1275.2	254.2	746.0
24	J	31/08/2021 21:26:53	b	In	46.148050	21.186227	NA	NA	NA	1980.0	3461.4	NA	NA	NA	NA	2785.6	NA	NA	NA	1263.1	242.1	759.9
25	J	31/08/2021 21:27:14	b	In	46.146394	21.184277	NA	NA	NA	2200.9	3676.5	NA	NA	NA	NA	3021.6	NA	NA	NA	1499.3	427.2	540.2
26	J	31/08/2021 21:27:36	b	Out	46.159026	21.195078	NA	NA	NA	1111.0	2473.5	NA	NA	NA	NA	1512.0	NA	NA	NA	427.5	**1237.8**	2153.7
27	J	01/09/2021 02:00:19	c	In	46.150049	21.185414	NA	NA	NA	NA	3433.2	NA	NA	NA	NA	2686.7	NA	NA	NA	1174.2	11.2	956.5
28	J	02/09/2021 21:44:03	d	Out	46.159778	21.192919	NA	NA	NA	NA	NA	NA	NA	NA	NA	NA	NA	NA	**1965.2**	596.5	1218.2	2163.6
29	J	02/09/2021 21:55:50	e	Out	46.162145	21.194494	NA	NA	NA	NA	NA	NA	NA	NA	NA	NA	NA	NA	**2220.3**	757.4	1507.8	2453.2
30	J	02/09/2021 21:55:58	e	Out	46.161530	21.194083	NA	NA	NA	NA	NA	NA	NA	NA	NA	NA	NA	NA	**2153.2**	707.8	1432.5	2378.0
31	J	02/09/2021 22:39:16	f	Out	46.136288	21.187019	NA	NA	NA	NA	NA	NA	NA	NA	NA	NA	NA	NA	924.9	2338.6	1548.0	679.8
32	J	02/09/2021 22:39:17	f	Out	46.137366	21.187594	NA	NA	NA	NA	NA	NA	NA	NA	NA	NA	NA	NA	810.0	2210.8	1433.1	603.5
33	J	02/09/2021 22:39:21	f	Out	46.136489	21.187334	NA	NA	NA	NA	NA	NA	NA	NA	NA	NA	NA	NA	891.9	2309.0	1527.9	672.6
34	J	02/09/2021 22:39:34	f	In	46.142859	21.192803	NA	NA	NA	NA	NA	NA	NA	NA	NA	NA	NA	NA	215.2	1501.6	991.8	783.2
35	J	02/09/2021 22:39:46	f	In	46.142770	21.192707	NA	NA	NA	NA	NA	NA	NA	NA	NA	NA	NA	NA	219.1	1513.3	995.8	774.2
36	J	02/09/2021 22:39:55	f	Out	46.148211	21.194511	NA	NA	NA	NA	NA	NA	NA	NA	NA	NA	NA	NA	671.7	**893.4**	734.4	1161.6
37	J	02/09/2021 22:40:01	f	In	46.148415	21.194397	NA	NA	NA	NA	NA	NA	NA	NA	NA	NA	NA	NA	695.3	874.7	719.5	1169.3
38	J	02/09/2021 22:40:12	f	In	46.148406	21.194668	NA	NA	NA	NA	NA	NA	NA	NA	NA	NA	NA	NA	692.3	868.9	739.9	1184.7
39	J	02/09/2021 22:40:19	f	In	46.143278	21.192968	NA	NA	NA	NA	NA	NA	NA	NA	NA	NA	NA	NA	224.1	1453.3	961.7	804.9
40	J	02/09/2021 22:40:40	f	In	46.147738	21.195093	NA	NA	NA	NA	NA	NA	NA	NA	NA	NA	NA	NA	615.9	931.1	793.6	1165.4
41	J	02/09/2021 22:40:45	f	In	46.147301	21.194630	NA	NA	NA	NA	NA	NA	NA	NA	NA	NA	NA	NA	570.1	987.5	778.6	1108.3
42	J	02/09/2021 22:40:53	f	Out	46.147602	21.196103	NA	NA	NA	NA	NA	NA	NA	NA	NA	NA	NA	NA	602.5	**929.4**	872.0	1221.4
43	J	03/09/2021 05:54:31	g	Out	46.138909	21.195667	NA	NA	NA	NA	NA	NA	NA	NA	NA	NA	NA	NA	367.9	1893.3	1480.5	1038.3
